# Direct and indirect mapping of the 12-item Short Form Survey version 2 (SF-12v2) onto the EQ-5D-5L utility scores in general Thai population

**DOI:** 10.1371/journal.pone.0351064

**Published:** 2026-06-22

**Authors:** Krittaphas Kangwanrattanakul

**Affiliations:** Division of Social and Administrative Pharmacy, Faculty of Pharmaceutical Sciences, Burapha University, 169 Long-Hard Bangsaen Rd, Mueang, Chonburi, Thailand; AstraZeneca Pharmaceuticals LP, UNITED STATES OF AMERICA

## Abstract

The SF-12v2 is widely used to measure health-related quality of life (HRQoL) in the general Thai population because it is brief and places a low burden on respondents. However, it does not provide utility scores required for economic analyses. The mapping approach is a solution to estimate the utility scores from the SF-12v2 response using several regression models. Therefore, this study aimed to develop a mapping algorithm to estimate EQ-5D-5L utility scores from the SF-12v2 items using a 2022 national dataset of 2000 Thai respondents. Four predictor sets incorporating SF-12v2 items/subscales and age as a covariate were investigated using direct and indirect mapping approaches. Direct mapping approaches included ordinary least squares, Tobit, censored least absolute deviations, generalized linear model (GLM), two-part models, adjusted limited dependent variable mixture model (ALDVMM), and beta mixture model, while multinomial logistic regression (MLOGIT) was investigated for indirect mapping. Model performance was evaluated using ten-fold cross-validated (post-CV) mean absolute error (MAE) and root mean square error (RMSE). The ALDVMM-1 component with a predictor set comprising age and selected SF-12 items as categorical variables demonstrated the best predictive performance, yielding the lowest post-CV MAE and RMSE (0.0410 and 0.0625, respectively). However, MLOGIT showed poorer predictive performance. Therefore, the proposed ALDVMM-1 component-based mapping algorithm may facilitate the estimation of utility scores in studies where only the SF-12v2 was collected. Given that the mapping algorithm was developed from a predominantly healthy sample, it may be less reliable in populations with poorer health status, and further validation in populations with less healthy or clinical samples is warranted in future studies.

## Introduction

Health technology assessment (HTA) is widely used to evaluate and identify the most cost-effective health interventions to support decision-making in healthcare [1]. It also optimizes the allocation of limited health resources [2,3]. Several HTA guidelines recommend cost-utility analysis to guide clinical decisions and policy implementation [4–6]. This approach compares incremental health improvement, expressed in quality-adjusted life years (QALYs), across or within health conditions [7]. QALYs are calculated using utility scores and life expectancy, making accurate measurement of utility scores essential [7,8].

Utility scores range from 0 (death or worst health state) to 1 (perfect health) [9]. Preference-based instruments, such as the EuroQol 5-dimension (EQ-5D), Health Utility Index (HUI), and Short Form 6 Dimension (SF-6D), measure these scores, which are then converted using a country-specific value set [10,11]. The EQ-5D-5L is the most commonly used instrument in Thailand. However, it may not capture some dimensions considered important, such as social health [12,13], and shows limited responsiveness to changes in both general [14] and patient populations [15].

Although preference-based instruments are standard for economic analyses, they do not provide dimension-specific (health profile) scores. Health profile instruments, such as the World Health Organization Quality of Life: Brief Version (WHOQOL-BREF) and the 12-item Short Form Survey version 2 (SF-12v2), offer more sensitivity to health changes [16,17]. However, they do not generate a utility score. Mapping between preference-based and profile instruments allows retention of profile information while producing utility scores for economic analyses.

Several Thai studies have mapped WHOQOL-BREF to EQ-5D-5L [18,19]. Although WHOQOL-BREF captures physical, psychological, social, and environmental health dimensions, its 26 items may still impose a respondent burden. The SF-12v2, derived from SF-36, is shorter, less burdensome, and widely used in large population surveys, including in Thailand [20–22]. Its conceptual overlap with EQ-5D-5L enhances suitability for mapping algorithms [14].

Despite its widespread use, SF-12v2 lacks a Thai-specific value set for producing utility scores. Mapping approaches can predict EQ-5D-5L utility scores from SF-12v2 responses using regression models [23,24]. Previous studies mapped SF-12v2 to the earlier EQ-5D-3L [25–31], which has a higher ceiling effect and lower discriminative power than EQ-5D-5L [32]. Ordinary least squares (OLS) models were sometimes used [26,27,29], but they are suboptimal for bounded utility scores with high ceiling effects and predict the utility scores outside the feasible range [33,34]. Alternative models, including Tobit, Beta mixture model (Betamix), Censored Least Absolute Deviation (CLAD), Generalized linear model (GLM), Adjusted Limited Dependent Variable Mixture Model (ALDVMM), and two-part models (TPM), better accommodate utility score characteristics [34–37].

To date, no Thai studies have mapped SF-12v2 to EQ-5D-5L using these models, nor performed indirect (response-based) mappings. This study aims to develop a mapping algorithm between SF-12v2 and EQ-5D-5L, including indirect mapping based on SF-12v2 responses in the general Thai population.

## Methods

### Study design and samples

This study used data from the project “EQ-5D-3L and EQ-5D-5L population norms in Thailand [38].” A cross-sectional survey was conducted using face-to-face interviews with 2,000 Thai adults. To ensure national representation, a four-stage stratified random sampling method was applied to select provinces, districts, sub-districts, and villages. Data were collected from 12 provinces, representing all geographic regions of Thailand: Bangkok, Samut Prakan, Nonthaburi, Chonburi, Nakhon Pathom, Chiang Mai, Nakhon Sawan, Nakhon Ratchasima, Khon Kaen, Buriram, Nakhon Si Thammarat, and Phatthalung.

Participants were eligible if they (1) were aged 18 years or older, (2) were able to understand the interview process as assessed by the researcher or trained interviewers, and (3) were fluent in Thai. Individuals with acute or life-threatening illnesses or severe cognitive impairments were excluded.

### Data collection procedures

Trained interviewers read all questions and response options verbatim, and did not provide explanations or interpretations to minimize interviewer bias. All participants completed both the SF-12v2 and EQ-5D-5L questionnaires, with no missing responses. These face-to-face interviews with eligible participants were conducted at their home residences between 1^st^ May to 30^th^ June 2023.

Before data collection, participants received an information sheet outlining study objectives, procedures, and their rights to withdraw without consequences. Written informed consent was obtained from all participants. Questionnaires were administered in the following order: (1) demographic information, (2) EQ-5D-5L, and (3) SF-12v2. Details of the demographic variables are reported elsewhere [38]. The protocol and data collection process were approved by the ethical committees of Burapha University Institutional Review Board (IRB1–031/2566) and adhered to the principles outlined in the Declaration of Helsinki.

### Source and target measures

#### SF-12v2 (source measure).

The SF-12v2 consists of 12 items grouped into eight subscales: physical functioning (PF: 2 items), role limitations due to physical problems (RP, 2 items), bodily pain (BP, 1 item), general health (GH, 1 item), vitality (VT, 1 item), social functioning (SF, 1 item), role limitations due to emotional problems (RE, 2 items), and mental health (MH, 2 items) [39]. Participants rated their health over a four-week recall period. Most items use a five-point Likert scale, except PF items, which use a three-point scale. Four items (GH1, BP1, VT1, MH1) were reverse-coded to ensure scoring consistency. Table 1 displays descriptions of the SF-12v2 items.

**Table 1:** Descriptions of the SF-12v2 items.

Item No.	Subscales	Item descriptions
1	General health (GH)	Health rating in general (GH1)
2	Physical functioning (PF)	Limitations in performing moderate activities (PF1)
3	Physical functioning (PF)	Limitations in climbing several flights of stairs (PF2)
4	Role limitations due to physical problems (RP)	Accomplished less due to physical problems (RP1)
5	Role limitations due to physical problems (RP)	Limited in kind of work due to physical problems (RP2)
6	Role limitations due to emotional problems (RE)	Accomplished less due to emotional problems (RE1)
7	Role limitations due to emotional problems (RE)	Not careful in work or activities due to emotional problems (RE2)
8	Bodily pain (BP)	Pain interfering with work inside or outside home (BP1)
9	Mental health (MH)	Feeling calm and peaceful (MH1)
10	Vitality (VT)	Having a lot of energy (VT1)
11	Mental health (MH)	Feeling downhearted and blue (MH2)
12	Social functioning (SF)	Physical or emotional problems interfere with social activities (SF1)

Subscale scores were transformed to a 0–100 scale, with higher scores indicating better health [39]. The Physical Component Summary (PCS) and Mental Component Summary (MCS) scores were calculated using a proprietary scoring algorithm to generate norm-based scores. SF-12v2 responses were also reclassified to derive SF-6D health states, allowing utility score estimation using the UK algorithm, with scores ranging from 0.29 to 1.00 [40].

#### EQ-5D-5L (target measure).

The EQ-5D-5L comprises five dimensions: mobility, self-care, usual activities, pain/discomfort, and anxiety/depression. Each dimension is assessed across five ordered severity levels: no problems, slight problems, moderate problems, severe problems, extreme problems/unable to perform. Responses across the five dimensions generate a five-digit health-state profile that characterizes an individual’s health status. These profiles are subsequently converted into utility scores using country-specific valuation algorithms.

In this study, EQ-5D-5L utility scores were derived using the Thai-specific value set, with values ranging from −0.4212, corresponding to the worst health state (55555), to 1.0000 representing full health (11111). The second-best health state (11121) is valued at 0.9436 [11].

The WHOQOL-BREF instrument was additionally employed to compute overall quality-of-life scores by summing four domain scores: physical health (7 items), psychological health (6 items), social relationships (3 items), and environment (8 items), using the ordinal-to-interval conversion table for general Thai population [41]. WHOQOL-BREF scores range from 24 to 120, with higher scores indicating better perceived health-related quality of life. These total scores were used to stratify participants and to plot mean EQ-5D-5L utility scores, facilitating comparisons between predicted and observed utility values.

### Conceptual overlap between EQ-5D-5L and SF-12v2

Conceptual overlap between the EQ-5D-5L and the SF-12v2 was examined using Spearman’s rank-order correlation analysis. Pairwise correlation coefficients (r) were calculated between each SF-12v2 item and subscale score and each EQ-5D-5L dimension, as well as the overall EQ-5D-5L utility score.

An SF-12v2 item or subscale demonstrating at least a moderate correlation (r ≥ 0.4) with any EQ-5D-5L dimension or the utility scores was deemed to exhibit conceptual overlap [42]. Items and subscales meeting the criterion were subsequently selected as candidate independent variables for inclusion in the predictor set of the regression models.

### Modeling approaches

Table 2 shows four predictor sets based on SF-12v2 dimensions and items in both direct and indirect mapping approaches. The explanatory variables were initially screened based on Spearman’s correlation between the SF-12v2 items/subscale scores and utility scores. The explanatory variables with p < 0.25 in the forward stepwise regression were included in the predictor sets. Furthermore, age was included as a covariate in all models due to its significant association with EQ-5D-5L utility scores and to reduce potential confounding effects. Accordingly, four predictor sets comprising subscale scores, polynomial transformation of subscale scores, and selected SF-12v2 items modeled as either continuous or categorical variables were evaluated using both mapping approaches.

**Table 2:** Four predictors sets based on SF-12v2 dimensions and items for direct and indirect mapping.

Set	SF-12v2 based predictors	Covariate
1	Subscale scores for physical functioning, role limitations due to physical problems, bodily pain, general health, and role limitations due to emotional problems	Age
2	Polynomial forms of the subscale scores for physical functioning, role limitations due to physical problems, bodily pain, general health, and role limitations due to emotional problems modeled as continuous variables	Age
3	Selected items from the 6 subscales: general health (item 1), physical functioning (items 2 and 3), role limitations due to physical problems (item 4), role limitations due to emotional problems (item 7), mental health (item 11) and social functioning (item 12) modeled as continuous variables	Age
4	Selected items from the 6 subscales: general health (item 1), physical functioning (item 2 and 3), role limitations due to physical problems (item 4), role limitations due to emotional problems (item 7), bodily pain (item 8), and social functioning (item 12) modeled as categorical variables	Age

A direct mapping approach was applied to predict EQ-5D-5L utility scores from SF-12v2 items and subscale-level scores. Multiple regression models were estimated, including OLS, Tobit, CLAD, GLM, TPM, ALDVMM, and Betamix. These models were selected based on their capacity to accommodate key features of EQ-5D-5L utility data, including boundedness, skewness, multimodality, and the discontinuity between the second-best and full-health states inherent in the Thai-specific value set.

In all direct mapping models, the EQ-5D-5L utility score served as the dependent variable, while SF-12v2 item/subscale scores and participant age were included as independent variables. For GLM and TPM specifications, disutility scores (1 – utility) were modeled to satisfy the non-negativity assumption of these approaches [43,44].

The modified Park test indicated that both Poisson and Gamma variance functions were plausible for the disutility outcome, as the estimated Gamma coefficient lay between 1 and 2 [45]. Based on Box-Cox regression results, a logarithmic link function was identified as optimal for both GLM and TPM models across all predictor sets [46].

Tobit and CLAD models were estimated to account for right-censoring, with utility scores censored at the upper bound of 1.0000 [31]. In contrast, ALDVMM and beta mixture models explicitly accommodated the multimodal distribution of utility scores while constraining predictions to the feasible utility range [37]. Accordingly, these models imposed lower and intermediate constraints at −0.4212 (worst health state) and 0.9436 (second-best health state), with truncation at 1.0000 for full health. Both mixture models were restricted to single-component specification, as models with additional components failed to converge across most predictor sets.

For the indirect mapping approach, each EQ-5D-5L dimension was modeled separately as a dependent variable, with SF-12v2 item/subscale scores serving as predictors. To estimate response-level probabilities, generalized ordered logistic (GOLOGIT), ordinal logistic (OLOGIT), or multinomial logistic (MLOGIT) models were evaluated.

GOLOGIT models failed to converge, and OLOGIT models were deemed inappropriate due to violations of the proportional odds assumption, indicating that predictor effects varied across response levels. Consequently, MLOGIT was selected as the final modeling strategy.

Using the most-likely-probability method, MLOGIT generated the most probable response level for each EQ-5D-5L dimension, producing a five-digit health-state profile that was subsequently converted into a utility score using the Thai-specific value set.

Notably, predicted utility scores were truncated at the theoretical boundaries of the Thai EQ-5D-5L value set when estimated values exceeded the feasible utility range. Specifically, values greater than 1 were truncated to the upper bound of 1.0, while values less than –0.4212 were truncated to the lower bound of –0.4212.

### Model performance

Model performance was evaluated using ten-fold cross-validation (CV), whereby the dataset was randomly partitioned into 10 approximately equal-sized subsets (n ≈ 200 per fold). In each iteration, nine subsets were used for model estimation, and the remaining subset served as the validation sample. This procedure was repeated 10 times so that each subset functioned as the validation set exactly once.

Predictive accuracy was assessed using the mean absolute error (MAE) and root mean square error (RMSE) across all predictor sets. The optimal model was identified based on the lowest values of these metrics, where discrepancies arose, RMSE was prioritized due to its greater sensitivity to large prediction errors [44].

Model agreement was further evaluated using the intraclass correlation coefficient (ICC) derived from a two-way mixed-effects model with absolute agreement and single-measurement specifications. ICC values were interpreted as poor (ICC < 0.50), moderate (0.50 ≤ ICC <0.75), good (0.75 ≤ ICC <0.90), or excellent (ICC ≥ 0.90) agreement [47].

In accordance with NICE guidelines [48], mean utility score plots were constructed across the full range of WHOQOL-BREF total scores to identify systematic prediction bias. Additionally, cumulative distribution plots were used to examine discrepancies between predicted and observed utility values. The optimal model was expected to demonstrate the highest agreement and minimal divergence across these visual diagnostics. Notably, this study was conducted and reported in accordance with the Mapping onto Preference-Based Measures Reporting Standards checklist [49] and the reporting standards guidance outlined in the 2017 by International Society for Pharmacoeconomics and Outcomes Research (ISPOR) Task Force Report [50], as throuroghly described in Tables in S1-S2 Table.

All statistical analyses were conducted using Stata version 17 (StataCorp LLC, College Station, TX, USA) and Microsoft Excel. A two-sided p-value <0.05 was considered statistically significant.

## Results

### Participant characteristics

Among the 2,000 participants drawn from the general Thai population, mean SF-12v2 subscale scores ranged from 64.07 ± 24.94 for GH to 86.83 ± 17.89 for RE. The mean PCS and MCS scores were 50.28 ± 8.44 and 53.61 ± 7.11, respectively. Most SF-12v2 subscales exhibited both ceiling effects (scores of 100) and floor effects (scores of 0). An exception was observed for the MH subscale, which demonstrated only a ceiling effect, affecting 14.50% of respondents. Detailed descriptive statistics for participant characteristics and EQ-5D-5L utility scores are reported elsewhere [38].

### Conceptual overlap between EQ-5D-5L and SF-12v2

Table 3 presents the absolute Spearman’s rank correlation coefficients between SF-12v2 items and subscale-level scores and EQ-5D-5L utility scores. All pairwise correlations were statistically significant (p < 0.001), except for the correlation between MH1 and SC, MCS, and MO, and MCS and SC.

**Table 3:** Spearman’s correlation coefficients between SF-12v2 items or subscale scores and the EQ-5D-5L dimensions and utility scores.

	EQ-5D-5L dimensions					
SF-12v2 items	MO	SC	UA	PD	AD	EQ-5D-5L utility scores
Health rating in general (GH1)	0.4156	0.2256	0.4714	0.5588	0.4006	0.6109
Limitations in performing moderate activities (PF1)	0.5107	0.3437	0.6148	0.5053	0.2900	0.5715
Limitations in climbing several flights of stairs (PF2)	0.5220	0.3179	0.5650	0.5115	0.2700	0.5664
Accomplished less due to physical problems (RP1)	0.4210	0.2881	0.5358	0.5418	0.3868	0.5994
Limited in kind of work due to physical problems (RP2)	0.4335	0.2981	0.5629	0.5256	0.3807	0.5914
Accomplished less due to emotional problems (RE1)	0.2335	0.2023	0.3376	0.3147	0.6876	0.5165
	**EQ-5D-5L dimensions**					
**SF-12v2 items**	**MO**	**SC**	**UA**	**PD**	**AD**	**EQ-5D-5L utility scores**
Not careful in work or activities due to emotional problems (RE2)	0.2551	0.2114	0.3637	0.3419	0.6725	0.5307
Pain interfering with work inside or outside home (BP1)	0.4336	0.2667	0.5261	0.5751	0.3695	0.6070
Feeling calm and peaceful (MH1)	0.0289	0.0139	0.0798	0.1286	0.2065	0.1837
Feeling downhearted and blue (MH2)	0.2547	0.1967	0.3811	0.3421	0.6466	0.5409
Having a lot of energy (VT1)	0.2239	0.1102	0.1639	0.2575	0.2083	0.2879
Physical or emotional problems interfere with social activities (SF1)	0.2140	0.1591	0.3625	0.3681	0.4217	0.4343
	**EQ-5D-5L dimensions**					
**SF-12v2 Subscales**	**MO**	**SC**	**UA**	**PD**	**AD**	**EQ-5D-5L utility scores**
PF	0.5318	0.3280	0.5965	0.5287	0.2881	0.5904
RP	0.4356	0.2957	0.5552	0.5488	0.3912	0.6107
BP	0.4336	0.2667	0.5261	0.5751	0.3695	0.6070
GH	0.4156	0.2256	0.4714	0.5588	0.4006	0.6109
VT	0.2239	0.1102	0.1639	0.2575	0.2083	0.2879
SF	0.2140	0.1591	0.3625	0.3681	0.4217	0.4343
RE	0.2446	0.2065	0.3460	0.3267	0.7005	0.5327
MH	0.1725	0.1083	0.2779	0.3007	0.5188	0.4457
PCS	0.5006	0.2984	0.5640	0.6024	0.2514	0.5986
MCS	0.0479	0.0464	0.1316	0.1912	0.5375	0.3312

All Spearman’s coefficients are shown in absolute values *AD* anxiety/depression *BP* bodily pain *GH* general health perception *MCS* mental component summary *MH* mental health *MO* mobility *PCS* physical component summary *PD* pain/discomfort *PF* physical functioning *RE* role limitation due to emotional problems *RP* role limitation due to physical problems *SC* self-care *SF* social functioning *UA* usual activity *VT* vitality

At the subscale level, PF, RP, BP, and GH demonstrated the strongest correlations with EQ-5D-5L utility scores (|r| = 0.5904–0.6109) and showed moderate to strong correlations with at least one EQ-5D-5L dimension. These subscales were therefore retained for inclusion in the predictor sets used in the mapping models. In contrast, VT exhibited the weakest correlation with EQ-5D-5L utility scores (|r| = 0.2879) and was excluded from further analyses.

At the item level, GH1, PF1, PF2, RP1, RP2, RE1, RE2, BP1, and MH2 showed at least moderate correlations with EQ-5D-5L utility scores (|r| > 0.4). However, the inclusion of RP1, RP2, RE1, and RE2 concurrently raised concerns regarding multicollinearity, given their conceptual and statistical overlap within subscales. Consequently, RP2 and RE1 were excluded from the predictor sets, as their correlation coefficients were lower than those of RP1 and RE2, respectively.

### Model selection

Table 4 summarizes predictive performance metrics across all regression models for both direct and indirect mapping approaches. For direct mapping, predictor set 4 demonstrated superior performance relative to the other sets, yielding the lowest average MAE and RMSE.

**Table 4:** Predictive performance of all regression models for predicting EQ-5D-5L utility scores from ten-fold cross-validation.

Models	Utility scores*			10-fold cross-validation		ICCs
	Mean	Minimum	Maximum	MAE	RMSE	
Observed	0.9230	−0.2216	1.0000	-	-	-
**Direct mapping**						
**Predictor set 1**						
OLS	0.9205	0.5537	1.0000	0.0444	0.0736	0.7236
Tobit	0.9392	0.4157	1.0000	0.0447	0.0762	0.7412
CLAD	0.9283	0.6129	1.0000	0.0442	0.0754	0.6773
GLM-gamma	0.8836	−0.4212	0.9941	0.0824	0.1739	0.5490
GLM-poisson	0.9230	−0.4212	0.9860	0.0495	0.0716	0.7764
TPM-gamma	0.9224	0.1029	0.9972	0.0454	0.0704	0.7614
TPM-poisson	0.9222	0.1077	0.9973	0.0452	0.0701	0.7691
ALDVMM-1	0.9212	0.4657	0.9963	0.0458	0.0717	0.7445
ALDVMM-2	0.9225	0.4909	0.9981	0.0452	0.0730	0.7306
Betamix	0.9234	−0.0586	0.9964	0.0482	0.0745	0.7522
**Predictor set 2**						
OLS	0.9225	0.3369	1.0000	0.0446	0.0700	0.7705
Tobit	0.9386	0.4317	1.0000	0.0434	0.0758	0.7551
CLAD	0.9295	0.5043	1.0000	0.0423	0.0737	0.7304
GLM-gamma	0.8915	−0.4212	0.9972	0.0719	0.1459	0.5645
GLM-poisson	0.9230	0.2705	0.9907	0.0482	0.0752	0.7506
TPM-gamma	0.9228	0.4054	0.9998	0.0431	0.0702	0.7687
TPM-poisson	0.9229	0.2552	0.9997	0.0431	0.0689	0.7825
ALDVMM-1	0.9220	0.4097	0.9957	0.0449	0.0691	0.7690
ALDVMM-2	Convergence not achieved					
Betamix	0.9239	0.1195	0.9997	0.0454	0.0743	0.7600
**Predictor set 3**						
OLS	0.9199	0.5751	1.0000	0.0441	0.0731	0.7274
Tobit	0.9386	0.4541	1.0000	0.0437	0.0749	0.7507
CLAD	0.9319	0.5731	1.0000	0.0434	0.0748	0.7306
GLM-gamma	0.8880	−0.4212	0.9960	0.0768	0.1619	0.5764
GLM-poisson	0.9230	−0.2246	0.9903	0.0486	0.0718	0.7804
TPM-gamma	0.9227	0.2956	0.9987	0.0444	0.0697	0.7665
TPM-poisson	0.9226	0.2785	0.9988	0.0441	0.0694	0.7751
ALDVMM-1	0.9212	0.4980	0.9985	0.0447	0.0706	0.7532
ALDVMM-2	0.9238	0.5304	0.9991	0.0442	0.0713	0.7380
Betamix	0.9233	0.1397	0.9983	0.0472	0.0742	0.7582
**Predictor set 4**						
OLS	0.9217	−0.2216	1.0000	0.0425	0.0670	0.8127
Tobit	0.9355	−0.2216	1.0000	0.0402	0.0697	0.8139
CLAD	0.9277	0.6041	1.0000	0.0445	0.0759	0.6816
GLM-gamma	0.9047	−0.4212	0.9977	Convergence not achieved		N/A
GLM-poisson	0.9230	−0.3909	0.9940	0.0442	0.0696	0.8126
TPM-gamma	0.9263	0.1060	0.9987	0.0431	0.0758	0.7096
TPM-poisson	0.9264	0.1938	0.9987	0.0432	0.0762	0.7083
ALDVMM-1	0.9222	−0.2214	0.9981	0.0410	0.0625	0.8192
ALDVMM-2	Convergence not achieved					
Betamix	Convergence not achieved					
**MLOGIT**						
Predictor set 1	0.9473	−0.4211	1.0000	0.0401	0.0703	0.7810
Predictor set 2	0.9387	−0.2216	1.0000	Convergence not achieved		N/A
Predictor set 3	Convergence not achieved					
Predictor set 4	Convergence not achieved					

*ALDVMM* adjusted limited dependent variable mixture model, *ALDVMM-1 part* ALDVMM with one component, *ALDVMM-2 part* ALDVMM with two components, *Betamix* beta regression-based mixture model, *CLAD* censored least absolute deviation, *GLM* generalized linear model, *ICC* Intraclass correlation coefficients, *MAE* mean absolute error, *MLOGIT* multinomial logistic regression, *OLS* ordinary least squares, *RMSE* root mean square error, *TPM* two-part models

*Mean, MAE and RMSE values were calculated after truncating predicted utility scores. The values greater than 1 and less than −0.4212 were set to 1 and −0.4212, respectively. MAE and RMSE were also calculated after performing 10-fold cross validation for all regression models.

GLM-gamma refers to a GLM estimated using a Gamma distribution with a log link function

GLM-poisson refers to a GLM estimated using a Poisson distribution with a log link function

TPM-gamma refers to a TPM in which the first part uses a logit function, and the second part is a GLM estimated with a Gamma distribution and a log link function.

TPM-poisson refers to a TPM, in which the first part uses a logit function, and the second part is a GLM estimated with a Poisson distribution and a log link function

Although both Tobit and ALDVMM-1 component models achieved the lowest post-cross-validation (CV) MAE (0.0402 and 0.0410, respectively), the ALDVMM-1 component model produced the lowest post-CV RMSE (0.0625). In contrast, the Tobit model ranked 16th in terms of RMSE among all evaluated models.

**Fig 1:**
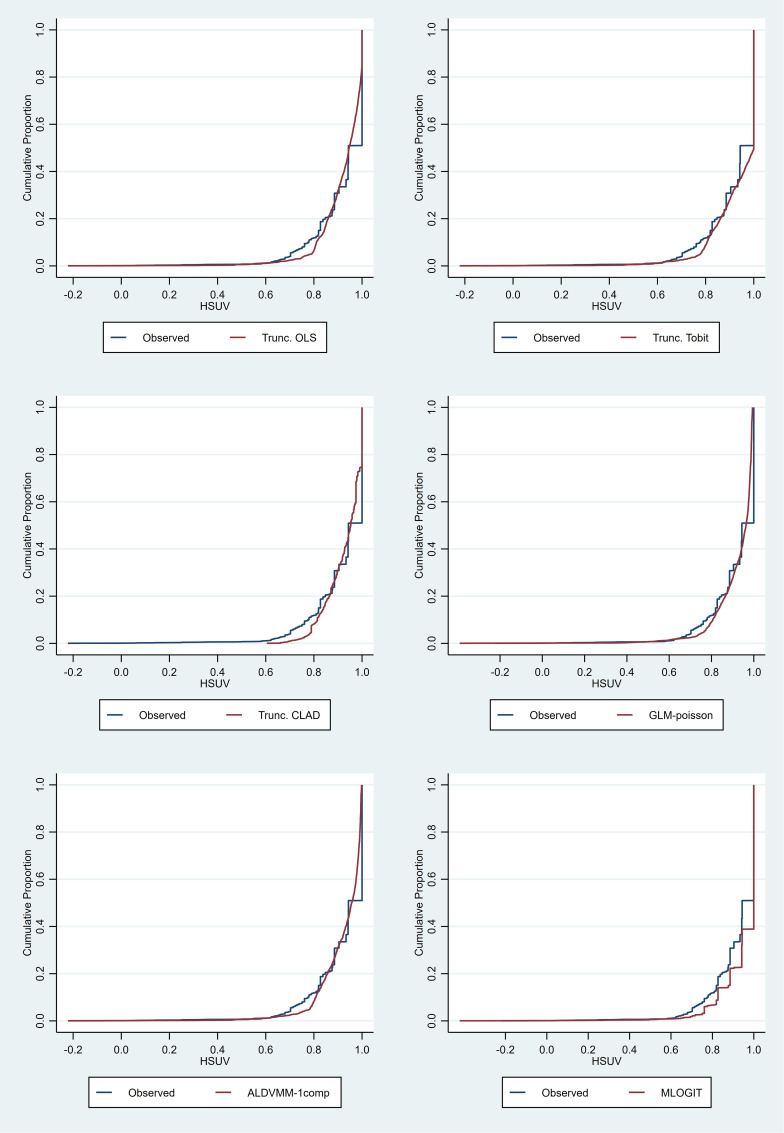
Comparison of the cumulative plots of observed and predicted utility scores across all regression models for direct and indirect mapping. *ALDVMM-1 component* adjusted limited dependent variable mixture model with 1 component *CLAD* censored least absolute deviation *GLM* generalized linear model *MLOGIT* multinomial logistic regression *OLS* ordinary least squares *Trunc* truncated predicted utility values for the OLS, Tobit, and CLAD models. Their plots were generated with predicted values truncated at theoretical boundaries of the Thai EQ-5D-5L value set: an upper bound of 1.0 for values exceeding 1 and a lower bound of –0.4212 for values less than –0.4212. Truncation did not affect the comparative assessment of model performance. Non-truncated predictions are shown for GLM-poisson, ALDVMM-1 component, and MLOGIT, which generate predicted utility values within the Thai-specific value set by model structures.

Based on overall predicted accuracy, the ALDVMM-1 component model was selected as the best-performing direct mapping model. As illustrated in Fig 1, this model exhibited less variability in predicted utility scores across the full distribution compared with the Tobit model. Although cumulative distribution plots indicated that the Tobit model more closely regressed observed values at the upper boundary (1.0000), this was primarily attributable to truncation at the maximum utility value. In contrast, the ALDVMM-1 component model generated predictions without truncation at either end of the scale. Similar to the distribution plots, ALDVMM-1 component model outperformed the other models because it could predict the utility scores more closely aligned with the observed utility scores, particularly at the maximum utility score of 1.00 (50%) and for values below zero as shown in Figure in S1 Fig.

Consistent with these findings, the ALDVMM-1 component model demonstrated the highest agreement between predicted and observed utility scores, with an ICC of 0.8192, exceeding that of all alternative regression models.

For indirect mapping, all five predictor sets were initially evaluated. However, the GOLOGIT model failed to converge, and the OLOGIT model violated the proportional odds assumption across all predictor sets. Consequently, MLOGIT was implemented.

Model convergence was achieved only for predictor set 1, yielding post-CV MAE and RMSE values of 0.0401 and 0.0703, respectively. Agreement between predicted and observed utility scores was good, with an ICC of 0.7810.

**Fig 2:**
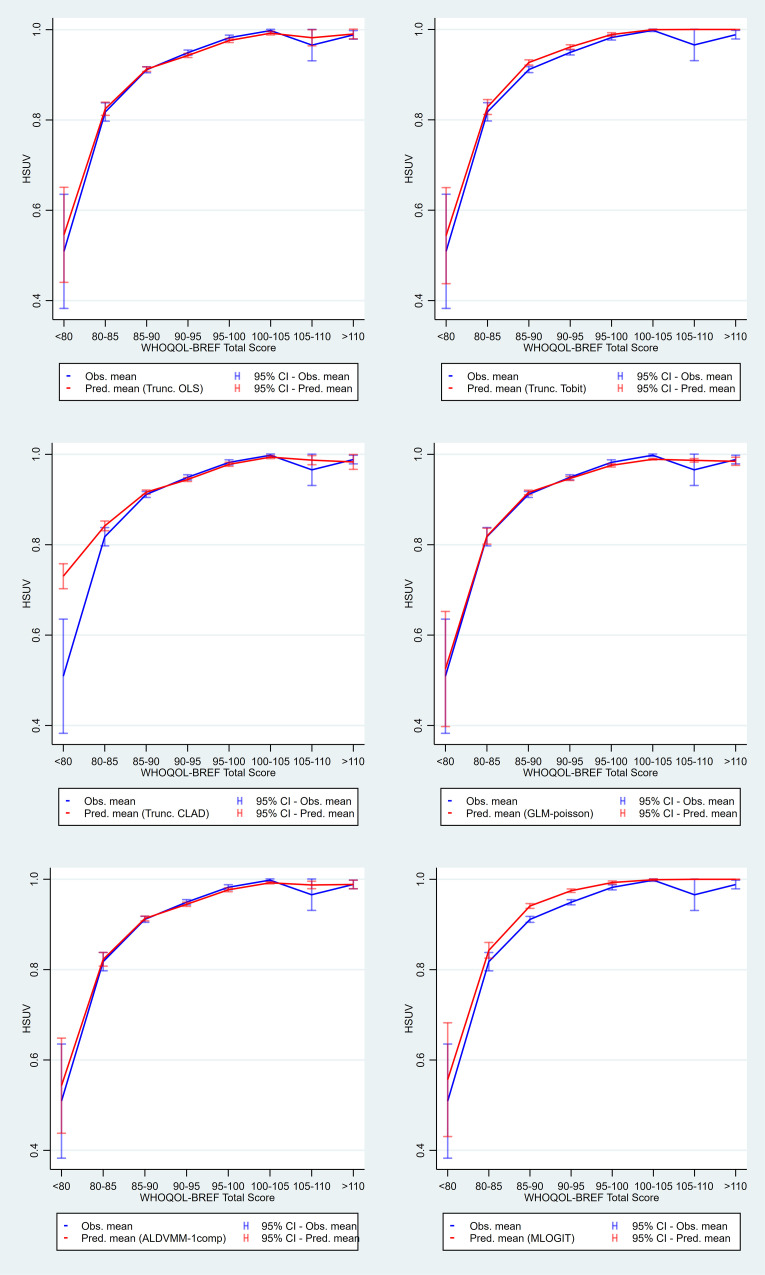
Mean observed and predicted utility scores by WHOQOL-BREF Total Score as the conditioning variable across all regression models for direct and indirect mapping. *ALDVMM-1 component* adjusted limited dependent variable mixture model with 1 component *CLAD* censored least absolute deviation *GLM* generalized linear model *MLOGIT* multinomial logistic regression *OLS* ordinary least squares *Trunc* truncated predicted utility values for the OLS, Tobit, and CLAD models. Their plots were generated with predicted values truncated at theoretical boundaries of the Thai EQ-5D-5L value set: an upper bound of 1.0 for values exceeding 1 and a lower bound of –0.4212 for values less than –0.4212. Truncation did not affect the comparative assessment of model performance. Non-truncated predictions are shown for GLM-poisson, ALDVMM-1 component, and MLOGIT, which generate predicted utility values within the Thai-specific value set by model structures.

Fig 2 presents plots comparing predicted and observed mean utility scores stratified by WHOQOL-BREF total scores across all regression models. All models exhibited a consistent upward trend in predicted utility scores with increasing WHOQOL-BREF scores. For WHOQOL-BREF scores below 80, the GLM-poisson produced the most accurate predictions, whereas other models tended to overestimate utility.

For WHOQOL-BREF scores between 80 and 105, the ALDVMM-1 component model demonstrated superior predictive performance compared to other regression models. In contrast, for scores exceeding 105, where data density was lower, the OLS model yielded more accurate predictions than other approaches. Notably, the MLOGIT model systematically overestimated utility scores across the entire WHOQOL-BREF score range.

### Algorithm for direct and indirect mapping

Based on the above evaluations, the ALDVMM-1 component model with predictor set 4 and the MLOGIT model with predictor set 1 were identified as the best-predicting models for direct and indirect mapping, respectively.

The mathematical specification for predicting EQ-5D-5L utility scores using the selected direct mapping model is shown below:


$$\begin{array}{c} \text{Estimated utility score }=\;[-\text{0}.\text{1309 }+\text{ age }(-\text{0}.\text{0011})\;+\;\sum_{i=\text{1}}^{n}\beta_{xi}.X_{i}] \end{array}$$

where:

($X_{i})$ denotes response to SF-12v2 items GH1, PF1, PF2, RP1, RE2, BP1, and SF1 (response levels for PF1 and PF2 ranged from 1–3; all others ranged from 1–5);

$\beta_{xi}$ represents the estimated coefficient corresponding to the item $\left(X_{i}\right);$

Age refers to the respondent’s age in years.

Estimated utility scores were subsequently used to calculate predicted utility values using algorithms derived from the StataCorp LLC implementation [51]. The predicted utility values were subsequently calculated using the following formula.


$$\begin{array}{c} \text{E}\left(\text{yi}|{\text{X}}^{\prime }\text{i}{\text{W}}^{\prime }\text{i}\right)=\;\sum_{c=\text{1}}^{C}\frac{\text{exp}({w^{\prime }}_{i}\delta_{c})}{\sum_{s=\text{1}}^{C}\text{exp}({w^{\prime }}_{i}\delta_{s})}\;[\left\{\text{1}-\phi \left(\frac{\psi_{\text{1}}-{X^{\prime }}_{i}\beta_{c}}{\sigma_{c}}\right)\right\}\;+\;\left\{\phi \left(\frac{\psi_{\text{1}}-{X^{\prime }}_{i}\beta_{c}}{\sigma_{c}}\right)\right\}\psi_{\text{2}}\;+\;\{\phi \left(\frac{\psi_{\text{1}}-{X^{\prime }}_{i}\beta_{c}}{\sigma_{c}}\right)\;-\;\phi \left(\frac{\psi_{\text{2}}-{X^{\prime }}_{i}\beta_{c}}{\sigma_{c}}\right)\} \end{array}$$

$$\begin{array}{c} \left\{{X^{\prime }}_{i}\beta_{c}+\sigma_{c}\frac{\emptyset \left(\frac{\psi_{\text{1}}-{X^{\prime }}_{i}\beta_{c}}{\sigma_{c}}\right)-\emptyset \left(\frac{\psi_{\text{2}}-{X^{\prime }}_{i}\beta_{c}}{\sigma_{c}}\right)}{\phi \left(\frac{\psi_{\text{2}}-{X^{\prime }}_{i}\beta_{c}}{\sigma_{c}}\right)-\phi \left(\frac{\psi_{\text{1}}-{X^{\prime }}_{i}\beta_{c}}{\sigma_{c}}\right)\;}\}\right] \end{array}$$

Where:

$\text{E}(\text{yi}|{\text{X}}^{\prime }\text{i}{\text{W}}^{\prime }\text{i})$: The predicted value of dependent variable yi (utility scores) for each individual

$\sum_{c=\text{1}}^{C}\frac{\text{exp}({w^{\prime }}_{i}\delta_{c})}{\sum_{s=\text{1}}^{C}\text{exp}({w^{\prime }}_{i}\delta_{s})}$: The summation operator over C possible categories; however, it is one for this study because the best-predicting model is ALDVMM with one component.

$\phi (\frac{\psi_{\text{1}}-{X^{\prime }}_{i}\beta_{c}}{\sigma_{c}})$: Cumulative distribution function of the standard normal distribution for upper limit

$\phi (\frac{\psi_{\text{2}}-{X^{\prime }}_{i}\beta_{c}}{\sigma_{c}})$: Cumulative distribution function of the standard normal distribution for lower limit

$\psi_{\text{2}}$: Upper bound is 0.9436 (the second highest values for the Thai-specific value set)

$\psi_{\text{2}}$: Lower bound is -0.4212 (the lowest values for the Thai-specific value set)

$\sigma_{c}$: The standard deviation of the error term for category c where it is 0.0795847 for

this study

${X^{\prime }}_{i}\beta_{c}$: Estimated utility value from the exploratory variable and regression coefficients

of the STATA output

$\emptyset (\frac{\psi_{\text{1}}-{X^{\prime }}_{i}\beta_{c}}{\sigma_{c}})$: Probability density function of the standard normal distribution for upper limit

$\emptyset (\frac{\psi_{\text{2}}-{X^{\prime }}_{i}\beta_{c}}{\sigma_{c}})$: Probability density function of the standard normal distribution for lower limit

Detailed coefficients and standard errors are provided in Table in S3 Table, with a worked example illustrated in Text in S1 Text.

Table in S4 Table reports the coefficients for the selected SF-12v2 items used in indirect mapping (predictor set 1), while Text in S2 Text provides a step-by-step example of utility scores calculated via the indirect mapping approach.

The predicted utility scores may be applied in future economic evaluations. The variance-covariance matrices are available in Microsoft Excel format to facilitate probabilistic sensitivity analysis in S2 File.

## Discussion

This study represents the first Thai mapping investigation to convert SF-12v2 responses into EQ-5D-5L utility scores using a Thai-specific value in a general population sample, employing both direct and indirect mapping approaches. In doing so, it addresses several methodological limitations of earlier mapping studies, many of which relied on EQ-5D-3L utilities [25–31] and predominantly applied OLS regression [26,27,29]. OLS is widely recognized as suboptimal for modeling health utility data because it fails to adequately accommodate ceiling effects and distributional irregularities [34].

Correlation analyses demonstrated substantial conceptual overlap between SF-12v2 items or subscale scores and EQ-5D-5L utility scores and dimensions. Most SF-12v2 subscales exhibited at least moderate correlations with EQ-5D-5L outcomes, except for the VT subscale and item. On this basis, the majority of SF-12v2 subscales and items were retained in the predictor sets. These findings are consistent with previous mapping studies conducted in both general and patient populations internationally, including studies from Thailand [14,52–54].

Although the MH subscale showed moderate correlations with AD and EQ-5D-5L utility scores, the MH1 item exhibited only weak correlations with EQ-5D-5L dimensions and utility. Moreover, inclusion of the MH subscale did not improve overall model fit. Consequently, only the MH2 item was retained in the final predictor set. PCS and MCS were excluded from all predictor sets because their inclusions introduced multicollinearity with SF-12v2 subscales (VIF > 0.40) and did not improve predictive performance.

For direct mapping, predictor set 4, which comprised categorical SF-12v2 items (GH1, PF1, PF2, RP1, RE2, BP1, and SF1), demonstrated the best predictive performance across models. Based on post-CV MAE and RMSE, the ALDVMM-1 component, OLS, and Tobit models emerged as the strongest candidates. However, OLS was excluded because it did not adequately account for the bounded, skewed, and heteroscedastic nature of utility data [55,56]. Although Tobit regression is theoretically suitable for censored outcomes, it did not yield the lowest model prediction errors, violated assumptions of normality and homoscedasticity [57], and produced predicted utility values exceeding 1.000, requiring artificial truncation. Consequently, the ALDVMM-1 component model was selected as the best direct mapping approach.

The ALDVMM-1 component model consistently demonstrated superior predictive performance. Its advantage lies in its ability to address key distributional challenges inherent in health utility data, including pronounced ceiling effects, multimodality, and the discontinuity between perfect health and the next-best health state [58]. These findings are consistent with previous mapping studies [25,37,58,59], indicating that mixture models outperform conventional linear models in both general and patient populations. Notably, the present ALDVMM-1 component outperformed earlier SF-12v2 mapping studies that relied on CLAD and simple OLS models based on PCS and MCS scores alone.

Although ALDVMM and beta mixture models are designed to accommodate the multimodality of utility distributions, the ALDVMM-2 component and beta mixture models failed to fully converge for the predictor set 4, which consisted of several SF-12v2 items coded as categorical variables in this study. This may be attributable to the substantial increase in the number of estimated parameters in these mixture models, resulting in instability and convergence difficulties. Furthermore, this mapping algorithm was developed from the predominantly healthy samples leading to sparse responses in certain categorical levels of the SF-12v2 items, particularly for poorer health status. Therefore, utility distribution might not have sufficient multimodality for complex mixture model specifications. ALDVMM-1 component can provide a more stable utility estimation while maintaining good predictive performance.

Whereas previous studies reported a mean prediction error of approximately 0.0744 and an MAE value around 0.14 in general [31] and socioeconomically disadvantaged US populations [27], the current study achieved markedly improved accuracy, with a mean error of 0.0008 and an MAE of 0.0410. These results support the inclusion of SF-12v2 items as exploratory variables rather than relying exclusively on summary component scores. Furthermore, the ALDVMM-1 component outperformed mixture models reported in earlier SF-12v2 mapping studies, despite those models being developed to predict EQ-5D-3L utility in US national samples. Specifically, the present model yielded a substantially lower post-CV RMSE (0.0625) compared with previously reported values ranging from 0.146 to 0.166 [25].

Because SF-12v2 items can be reclassified to derive the SF-6D instrument, the UK valuation algorithm is commonly applied in Thailand in the absence of a Thai-specific value set. Previous Thai studies have supported the use of SF-6D utilities for HRQoL measurement and economic evaluations in both the general [14] and chronic disease populations [52]. However, the present findings did not demonstrate conceptual overlap between the VT1 item and EQ-5D-5L utilities or dimensions. Moreover, agreement between SF-6D–derived and observed EQ-5D-5L utilities was lower (ICC = 0.7300) than that observed between predicted and observed EQ-5D-5L utilities using the proposed algorithm (ICC = 0.8192) with the general Thai samples in this present dataset.

Importantly, the proposed mapping algorithm showed greater sensitivity in poorer health states, predicting utility values as low as −0.2214. This value is substantially lower than the minimum utility score of 0.29 obtainable using the UK SF-6D algorithm [40]. In the absence of a Thai-specific SF-6D value set, the ALDVMM-based mapping equation is therefore recommended for estimating EQ-5D-5L utility scores from SF-12v2 data in general Thai populations.

For indirect mapping, the MLOGIT model with predictor set 1 is recommended. However, indirect mapping did not outperform direct mapping, as reflected by a higher post-CV RMSE (0.0703 vs 0.0625). Distributional and cumulative plots further indicated systematic overprediction of utility values above 0.6. Although OLOGIT is theoretically appropriate for ordinal outcomes [60], it was unsuitable in this study due to violations of the proportional odds assumption and convergence issues. Consequently, MLOGIT was employed to estimate EQ-5D-5L dimensions response probabilities.

The relatively weaker performance of indirect mapping may be attributable to sparse extreme responses within the EQ-5D-5L dimension in this generally healthy sample, leading to inflated standard errors. Similar challenges have been documented in previous mapping studies [30,44]. Despite these limitations, the MLOGIT model demonstrated superior performance compared with earlier US-based studies, achieving a lower MAE (0.0401 vs 0.0480) [30]. This improvement may reflect the enhanced discriminative capacity and reduced ceiling effects of the previous mapping study estimating the EQ-5D-3L utility scores, which are noted to have higher ceiling effects of EQ-5D-5L relative to EQ-5D-3L [30].

Although there are several mapping algorithm generated from general representative samples from other countries, they might not be applicable to Thai population because mapping algorithms are largely population-dependent because both predictor distributions and health-state valuations may vary across countries and cultural contexts. Differences in demographic composition and baseline health status between the present Thai sample and the other populations used to develop their country -based mapping algorithms may therefore influence model estimation and predictive accuracy. In the previous US-based mapping algorithm from the US representative samples [31], two key differences may contribute to the observed performance gap between the two algorithms. First, the US-derived algorithms were developed using a sample in which the majority of respondents presenting with more chronic conditions (average number of chronic conditions = 1.90), compared with approximately 0.37 in the Thai derivation sample. Therefore, the US-derived algorithms may predict utility scores more accurately among unhealthy respondents or those at the lower end of the health spectrum than the Thai mapping algorithm. This explanation is supported by the lower mean utility score estimated using the US-based algorithm (0.8912), compared with that estimated using the Thai mapping algorithm (0.9222). Second, cultural differences between these two countries can influence how respondents interpret and report HRQoL items because health perception is inherently shaped by sociocultural context. Previous study has demonstrated that certain culture-specific health dimensions perceived by the Thai population differ from those reported in Western populations [61]. As a result, these factors may help explain why a Thai-specific mapping algorithm is required for estimating utility scores in the Thai population. Nevertheless, the performance of the Thai mapping algorithm should be further validated using an independent dataset from the general Thai population, particularly among unhealthy individuals or clinical population.

Several limitations should be acknowledged. First, this study sample comprised predominantly healthy individuals from the general population, resulting in a sparse representation of severe health states across EQ-5D-5L dimensions. Consequently, the mapping algorithm may be less accurate for populations with poorer health. Second, although 10-fold CV was conducted to evaluate the model performance and reduce the overfitting of the mapping algorithm, the external validation using an independent dataset was not performed. Future studies should validate and refine this proposed mapping algorithm in less healthy samples or clinical population before broader application in economic analyses.

## Conclusions

This study developed a novel mapping algorithm to convert SF-12v2 responses into EQ-5D-5L utility scores for the general Thai population. While earlier Thai studies have supported the application of the UK valuation algorithm to derive SF-6D utility score, the present findings challenge this practice. Specifically, correlation analyses did not demonstrate sufficient conceptual overlap between the vitality subscale or item and EQ-5D-5L dimension or scores. In addition, agreement between SF-6D–derived and observed EQ-5D-5L utility scores was lower than that observed between the mapped and observed EQ-5D-5L utility generated by the proposed algorithm.

Despite these strengths, an important limitation should be acknowledged. The proposed mapping algorithm may have reduced accuracy in predicting utility scores among individuals with poor health status, reflecting the limited representation of severe health states in the study sample. Accordingly, caution is warranted when applying this algorithm to populations with substantial morbidity, and further validation in less healthy or clinical samples is recommended.

## Supporting information

S1 Table Guidelines and checklist for mapping onto Preference-Based Measures Standard (MAPS) checklist. (DOCX)

S2 Table Checklist for 2017 ISPOR Good Practices Report: Mapping to Estimate Health-State Utility from Non-Preference-Based Outcome Measures. Summary of reporting of mapping studies recommendations.
(DOCX)

S3 Table Coefficients and standard errors of the final model used for direct mapping based on the Thai value set. (DOCX)

S4 Table Coefficients and standard errors of the final model used for indirect mapping. (DOCX)

S1 Text Instructions for predicting utility scores using the direct mapping algorithm. (DOCX)

S2 Text Instructions for predicting utility scores using the indirect mapping algorithm. (DOCX)

S1 Fig Comparison of the distributions of observed and predicted utility scores across all regression models for direct and indirect mapping. (DOCX)

S1 File Data used in the study. (XLSX)

S2 File Variance-Covariance matrix for both direct and indirect mapping algorithms. (XLSX)
