# Supplementary material for: Direct and indirect mapping of the 12-item Short Form Survey version 2 (SF-12v2) onto the EQ-5D-5L utility scores in general Thai population
Source: PLoS One. 2026 Jun 22;21(6):e0351064. doi: 10.1371/journal.pone.0351064 (PMC13286156; doi:10.1371/journal.pone.0351064)
Supplement: S1 Table — (DOCX) [file pone.0351064.s001.docx]

**S1 Table. Guidelines and checklist for mapping onto Preference-Based Measures Standard (MAPS) checklist**

| **Section/topic** | **Item no.** | **Recommendation** | **Reported in** |
| --- | --- | --- | --- |
| **Title and abstract** |  |  |  |
| Title | 1 | Identify the report as a study mapping between outcome measures. State the source measure(s) and generic, preference-based target measure(s) used in the study. | Title |
| Abstract | 2 | Provide a structured abstract including, as applicable: objectives; methods, including data sources and their key characteristics, outcome measures used and estimation and validation strategies; results, including indicators of model performance; conclusions; and implications of key findings. | Abstract |
| **Introduction** |  |  |  |
| Study rationale | 3 | Describe the rationale for the mapping study in the context of the broader evidence base. | Introduction section: Paragraph 2, 3, 4, and 5 |
| Study objective | 4 | Specify the research question with reference to the source and target measures used and the disease or population context of the study. | Introduction section: the last sentence of paragraph 6 |
| **Methods** |  |  |  |
| Estimation sample | 5 | Describe how the estimation sample was identified, why it was selected, the methods of recruitment and data collection, and its location(s) or setting(s) | Methods: Study design and samples section |
| External validation sample | 6 | If an external validation sample was used, the rationale for selection, the methods of recruitment and data collection, and its location(s) or setting(s) should be described. | Absence of external dataset for external validation in this study. |
| Source and target measures | 7 | Describe the source and target measures and the methods by which they were applied in the mapping study. | Methods: Source and target measures section |
| Exploratory data analysis | 8 | Describe the methods used to assess the degree of conceptual overlap between the source and target measures. | Methods: Conceptual overlap between EQ-5D-5L and SF-12v2 section |
| Missing data | 9 | State how much data were missing and how missing data were managed in the sample(s) used for the analyses. | Methods: data collection process section. |
| Modelling approaches | 10 | Describe and justify the statistical model(s) used to develop the mapping algorithm. | Methods: Modelling approach section |
| Estimation of predicted scores or utilities | 11 | Describe how predicted scores or utilities are estimated for each model specification. | Methods: Paragraph 2,3,4,5,6, 7, 8 and 9 of the modelling approach |
| Validation methods | 12 | Describe and justify the methods used to validate the mapping algorithm. | Methods: Paragraph 1 of the model performance |
| Measures of model performance | 13 | State and justify the measure(s) of model performance that determine the choice of the preferred model(s) and describe how these measures were estimated and applied. | Methods: Paragraph 2 and 3 of the model performance |
| **Results** |  |  |  |
| Final sample size(s) | 14 | State the size of the estimation sample and any validation sample(s) used in the analyses (including both number of individuals and number of observations). | Result: Participant characteristics |
| Descriptive information | 15 | Describe the characteristics of individuals in the sample(s) (or refer back to previous publications giving such information). Provide summary scores for source and target measures, and summarize results of analyses used to assess overlap between the source and target measures. | Results: Descriptive statistics |
| Model selection | 16 | State which model(s) is(are) preferred and justify why this(these) model(s) was(were) chosen. | Results: Model selection |
| Model coefficients | 17 | Provide all model coefficients and standard errors for the selected model(s). Provide clear guidance on how a user can calculate utility scores based on the outputs of the selected model(s). | -Results: Algorithm for direct and indirect mapping  -Excel sheet for calculating the predicted utility scores for both direct and indirect mapping upon request.  -Supporting information (S1_Text, S2_Text, and S2_File) |
| Uncertainty | 18 | Report information that enables users to estimate standard errors around mean utility predictions and individual-level variability. |  |
| Model performance and face validity | 19 | Present results of model performance, such as measures of prediction accuracy and fit statistics for the selected model(s) in a table or in the text. Provide an assessment of face validity of the selected model(s). |  |
| **Discussion** |  |  |  |
| Comparisons with previous studies | 20 | Report details of previously published studies developing mapping algorithms between the same source and target measures and describe differences between the algorithms, in terms of model performance, predictions and coefficients, if applicable. | Discussion paragraph 3, 4, 5, 6, 7, 8, 9, 10, and 11. Please note that none of the previous studies mapped the SF-12v2 with the EQ-5D-5L. |
| Study limitations | 21 | Outline the potential limitations of the mapping algorithm. | Discussion paragraph 12 and 13 |
| Scope of applications | 22 | Outline the clinical and research settings in which the mapping algorithm could be used. | Discussion and conclusion |
| **Other** | | | |
| Additional information | 23 | Describe the source(s) of funding and non-monetary support for the study, and the role of the funder(s) in its design, conduct and report. Report any conflicts of interest surrounding the roles of authors and funders. | Declarations |
