# Supplementary material for: Direct and indirect mapping of the 12-item Short Form Survey version 2 (SF-12v2) onto the EQ-5D-5L utility scores in general Thai population
Source: PLoS One. 2026 Jun 22;21(6):e0351064. doi: 10.1371/journal.pone.0351064 (PMC13286156; doi:10.1371/journal.pone.0351064)
Supplement: S2 Table — Summary of reporting of mapping studies recommendations. (DOCX) [file pone.0351064.s002.docx]

**S2 Table. Checklist for 2017 ISPOR Good Practices Report: Mapping to Estimate Health-State Utility from Non-Preference-Based Outcome Measures. Summary of reporting of mapping studies recommendations**

| **No.** | **Recommendation** | **Reported in** |
| --- | --- | --- |
| 1. | Describe relevant differences between data sets that are candidates for mapping estimation | Not applicable because a single dataset was used in this study |
| 2 | Give full details of the selected data set. Describe how the study was run and patients were sampled. Provide baseline and follow up characteristics including the distribution of patients’ disease severity. Missingness in the longitudinal pattern of responses should be described. | Methods: Study design and samples. This study was conducted with general population, so the patients’ disease severity was not reported. |
| 3 | Plot the distribution of the utility data. | Supporting information: S1_Fig Plot the distribution of the observed utility and predicted utility scores from all regression models |
| 4 | Justify the type of model(s) selected with reference to the characteristics of the target utility distribution and the proposed use of the mapping function | Methods: Modelling approach |
| 5 | Compare the dimensions of health covered by the target utility instrument and those covered by the explanatory clinical measure(s) | Methods: Conceptual overlap between EQ-5D-5L and SF-12v2  in Table 3: Absolute correlation coefficients between EQ-5D-5L and SF-12v2 instruments |
| 6 | Describe the approach to determining the final model. Include tests conducted and judgments made. | Methods: Second and third paragraph in the Model performance section |
| 7 | Summary measures of fit are of limited value for the total sample. Provide information on fit conditional on disease severity as measured by the clinical outcome measure(s). A plot of mean predicted versus mean observed utility conditional on the clinical variable(s) should be included. | Figure 2: The plot between observed and predicted utility scores against WHOQOL-BREF Total score across all regression models for direct and indirect mapping  Table 4: Summary of model performance indices (MAE and RMSE) for each regression model |
| 8 | Coefficient values, error term(s) distributions(s), variances, and covariances are required. | Supporting information: S3_Table and S4_Table for direct and indirect mapping approaches  Results: Algorithm for direct and indirect mapping  Excel sheet for calculating the predicted utility scores for both direct and indirect mapping upon request. |
| 9 | Provide an example predicted value for some sets of covariates. Consider providing a program that calculates predictions for user-defined inputs. | Excel sheet for calculating the predicted utility scores for both direct and indirect mapping upon request.  Supporting information: S1_Text and S2_Text shows example of how to compute the utility scores using both approaches |
| 10 | Parameter uncertainty in a mapping regression should be reflected using standard methods for PSA. Assessment of model suitability for use in cost-effectiveness analysis should also consider the distribution of utility values for PSA, with particular focus on whether these lie outside the feasible utility range for the PBM | Supporting Information: S2_File |
| 11 | When imputing data from a mapping function, individual-level variability should be incorporated using simulation methods and information about the distribution of the error term(s). These simulated data can be compared with the raw observed data, including an assessment of the range of values compared with the feasible range for the PBM | Supporting Information: S2_File |
| 12 | Re-estimation of mapping results in a separate data set or other forms of validation are not routinely required. | Absence of external dataset for external validation in this study. |
