# Supplementary material for: Direct and indirect mapping of the 12-item Short Form Survey version 2 (SF-12v2) onto the EQ-5D-5L utility scores in general Thai population
Source: PLoS One. 2026 Jun 22;21(6):e0351064. doi: 10.1371/journal.pone.0351064 (PMC13286156; doi:10.1371/journal.pone.0351064)
Supplement: S3 Table — (DOCX) [file pone.0351064.s003.docx]

**S3 Table.** **Coefficients and standard errors of the final model used for direct mapping based on the Thai value set.**

| **Covariate** | **Coefficient** | **SE** |
| --- | --- | --- |
| Constant | -0.1309 | 0.0807 |
| Age | -0.0011 | 0.0002 |
| GH1 |  |  |
| 1 | 0.0000 | N/A |
| 2 | 0.1489 | 0.0195 |
| 3 | 0.1854 | 0.0200 |
| 4 | 0.2208 | 0.0205 |
| 5 | 0.2456 | 0.0226 |
| PF1 |  |  |
| 1 | 0.0000 | N/A |
| 2 | 0.0786 | 0.0161 |
| 3 | 0.1049 | 0.0176 |
| PF2 |  |  |
| 1 | 0.0000 | N/A |
| 2 | 0.0036 | 0.0135 |
| 3 | 0.0033 | 0.0152 |
| RP1 |  |  |
| 1 | 0.0000 | N/A |
| 2 | 0.4343 | 0.0424 |
| 3 | 0.4952 | 0.0430 |
| 4 | 0.4828 | 0.0431 |
| 5 | 0.5096 | 0.0431 |
| RE2 |  |  |
| 1 | 0.0000 | N/A |
| 2 | -0.0281 | 0.0620 |
| 3 | 0.0529 | 0.0591 |
| 4 | 0.0639 | 0.0592 |
| 5 | 0.1252 | 0.0593 |
| BP1 |  |  |
| 1 | 0.0000 | N/A |
| 2 | 0.0293 | 0.0279 |
| 3 | -0.0193 | 0.0267 |
| 4 | -0.0134 | 0.0268 |
| 5 | 0.0354 | 0.0271 |
| SF1 |  |  |
| 1 | 0.0000 | N/A |
| 2 | 0.2458 | 0.1105 |
| 3 | 0.2417 | 0.1092 |
| 4 | 0.2310 | 0.1093 |
| 5 | 0.2304 | 0.1093 |
