# Supplementary material for: Direct and indirect mapping of the 12-item Short Form Survey version 2 (SF-12v2) onto the EQ-5D-5L utility scores in general Thai population
Source: PLoS One. 2026 Jun 22;21(6):e0351064. doi: 10.1371/journal.pone.0351064 (PMC13286156; doi:10.1371/journal.pone.0351064)
Supplement: S4 Table — (DOCX) [file pone.0351064.s004.docx]

**S4 Table.** **Coefficients and standard errors of the final model used for indirect mapping**

| **Mobility (Level 1 is the reference/base group)** | | | | | | | | |
| --- | --- | --- | --- | --- | --- | --- | --- | --- |
|  | **Level 2** | | **Level 3** | | **Level 4** | | **Level 5** | |
| **Predictor** | **Coefficient** | **SE** | **Coefficient** | **SE** | **Coefficient** | **SE** | **Coefficient** | **SE** |
| Constant | -2.273 | 0.641 | -2.574 | 1.096 | -15.706 | 8.219 | 65.207 | 14710.630 |
| Age | 0.061 | 0.007 | 0.082 | 0.014 | 0.274 | 0.109 | 0.205 | 205.723 |
| PF | -0.024 | 0.004 | -0.022 | 0.006 | 0.014 | 0.027 | -0.559 | 87.758 |
| RP | 0.000 | 0.006 | -0.026 | 0.009 | 0.023 | 0.045 | -2.539 | 145.478 |
| BP | -0.004 | 0.005 | -0.025 | 0.008 | -0.078 | 0.064 | -1.259 | 71.326 |
| GH | -0.021 | 0.004 | -0.027 | 0.007 | -0.166 | 0.058 | -0.098 | 97.186 |
| RE | 0.003 | 0.005 | 0.013 | 0.007 | -0.026 | 0.026 | 0.024 | 33.701 |

| **Self-care (Level 1 is the reference/base group)** | | | | | | | | |
| --- | --- | --- | --- | --- | --- | --- | --- | --- |
|  | **Level 2** | | **Level 3** | | **Level 4** | | **Level 5** | |
| **Predictor** | **Coefficient** | **SE** | **Coefficient** | **SE** | **Coefficient** | **SE** | **Coefficient** | **SE** |
| Constant | -4.564 | 1.114 | -5.357 | 2.560 | -1.344 | 5.289 | -165.654 | 149336.800 |
| Age | 0.079 | 0.014 | 0.089 | 0.033 | 0.026 | 0.061 | 2.393 | 2230.491 |
| PF | -0.014 | 0.006 | -0.038 | 0.013 | -0.637 | 143.071 | 0.500 | 1670.675 |
| RP | -0.009 | 0.009 | -0.017 | 0.017 | -0.104 | 0.062 | -5.923 | 489.186 |
| BP | -0.003 | 0.008 | -0.018 | 0.015 | -0.021 | 0.030 | 1.050 | 42.896 |
| GH | 0.000 | 0.006 | -0.006 | 0.014 | 0.030 | 0.032 | 1.390 | 1518.144 |
| RE | -0.020 | 0.007 | -0.008 | 0.011 | 0.007 | 0.026 | -1.033 | 1987.027 |

| **Usual activities (Level 1 is the reference/base group)** | | | | | | | | |
| --- | --- | --- | --- | --- | --- | --- | --- | --- |
|  | **Level 2** | | **Level 3** | | **Level 4** | | **Level 5** | |
| **Predictor** | **Coefficient** | **SE** | **Coefficient** | **SE** | **Coefficient** | **SE** | **Coefficient** | **SE** |
| Constant | 2.624 | 0.609 | 3.062 | 1.102 | -0.799 | 2.941 | -68.816 | 6631.227 |
| Age | 0.040 | 0.007 | 0.055 | 0.013 | 0.079 | 0.034 | 1.142 | 87.572 |
| PF | -0.022 | 0.004 | -0.034 | 0.007 | -0.040 | 0.016 | -0.005 | 47.670 |
| RP | -0.025 | 0.006 | -0.053 | 0.010 | -0.060 | 0.026 | -2.381 | 104.983 |
| BP | -0.004 | 0.005 | -0.025 | 0.008 | -0.087 | 0.022 | 0.332 | 39.248 |
| GH | -0.023 | 0.004 | -0.031 | 0.007 | -0.017 | 0.016 | 0.228 | 69.299 |
| RE | -0.018 | 0.005 | -0.004 | 0.007 | 0.030 | 0.017 | -0.052 | 49.789 |

**S2 Table.** **Coefficients and standard errors of the final model used for indirect mapping (MLOGIT with set 1) (cont.)**

| **Pain/discomfort (Level 1 is the reference/base group)** | | | | | | | | |
| --- | --- | --- | --- | --- | --- | --- | --- | --- |
|  | **Level 2** | | **Level 3** | | **Level 4** | | **Level 5** | |
| **Predictor** | **Coefficient** | **SE** | **Coefficient** | **SE** | **Coefficient** | **SE** | **Coefficient** | **SE** |
| Constant | 3.376 | 0.501 | 4.854 | 0.678 | 3.068 | 1.985 | 11.193 | 5.646 |
| Age | 0.024 | 0.005 | 0.021 | 0.007 | 0.027 | 0.023 | -0.055 | 0.067 |
| PF | -0.009 | 0.003 | -0.013 | 0.004 | -0.025 | 0.012 | -0.061 | 0.041 |
| RP | -0.015 | 0.005 | -0.021 | 0.006 | -0.014 | 0.017 | -0.00000182 | 6.000 |
| BP | -0.007 | 0.004 | -0.022 | 0.005 | -0.061 | 0.015 | 0.033 | 0.055 |
| GH | -0.034 | 0.003 | -0.051 | 0.005 | -0.053 | 0.014 | -0.157 | 0.081 |
| RE | -0.007 | 0.004 | -0.004 | 0.005 | 0.009 | 0.013 | -0.126 | 0.055 |

| **Anxiety/depression (Level 1 is the reference/base group)** | | | | | | | | |
| --- | --- | --- | --- | --- | --- | --- | --- | --- |
|  | **Level 2** | | **Level 3** | | **Level 4** | | **Level 5** | |
| **Predictor** | **Coefficient** | **SE** | **Coefficient** | **SE** | **Coefficient** | **SE** | **Coefficient** | **SE** |
| Constant | 8.511 | 0.617 | 10.642 | 0.843 | 4.651 | 4.117 | 12.361 | 5.774 |
| Age | 0.003 | 0.005 | -0.011 | 0.008 | 0.047 | 0.048 | -0.026 | 0.078 |
| PF | 0.001 | 0.004 | 0.010 | 0.006 | 0.000 | 0.026 | 0.014 | 0.031 |
| RP | 0.002 | 0.005 | 0.001 | 0.008 | -0.022 | 0.038 | 0.067 | 0.048 |
| BP | 0.014 | 0.005 | 0.008 | 0.006 | 0.047 | 0.030 | 0.065 | 0.044 |
| GH | -0.028 | 0.004 | -0.040 | 0.005 | -0.010 | 0.027 | -0.174 | 0.069 |
| RE | -0.108 | 0.006 | -0.140 | 0.008 | -0.192 | 0.037 | -0.338 | 0.075 |
