# Supplementary material for: Direct and indirect mapping of the 12-item Short Form Survey version 2 (SF-12v2) onto the EQ-5D-5L utility scores in general Thai population
Source: PLoS One. 2026 Jun 22;21(6):e0351064. doi: 10.1371/journal.pone.0351064 (PMC13286156; doi:10.1371/journal.pone.0351064)
Supplement: S1 Text — (DOCX) [file pone.0351064.s005.docx]

**S1 Text. Instructions for predicting utility scores using the direct mapping algorithm**

**Example**: A 74-year-old respondent provided the following responses to the SF-12v2 items included as predictors in the direct mapping algorithm

| **Item** | **GH1** | **PF1** | **PF2** | **RP1** | **RE2** | **BP1** | **SF1** |
| --- | --- | --- | --- | --- | --- | --- | --- |
| Original response | 4 | 1 | 1 | 3 | 2 | 3 | 3 |
| **Recode** | **GH1_r** |  |  |  |  | **BP1_r** |  |
| Final response | 2 | 1 | 1 | 3 | 2 | 3 | 3 |

Please note that the response to GH1 and BP1 should be recoded prior to computing the predicted utility scores

**Step 1:** Calculate the predicted utility value by summing the corresponding coefficients for each SF-12v2 item ($\beta_{X_{i}}$), as provided in the Table in Supplementary Section 1 to obtain the total predicted utility scores.

Estimated utility scores

= $-$0.1309 + Age (β_age_) + $\beta_{GH1\_r}$+ $\beta_{PF1}$ + $\beta_{PF2}$ + $\beta_{RP1}$ + $\beta_{RE2}$ + $\beta_{BP1\_r}$ + $\beta_{SF1}$

= $-$0.1309 + 74 (-0.0011) + 0.1489 + 0.0000 + 0.0000 + 0.4952 $-$0.0281$-$ 0.0193 + 0.2417

= 0.6236482

**Step 2** Calculate the predicted utility values from the estimated utility value derived from Step 1 using the direct mapping algorithm addressed in the main manuscript

Predicted utility values = 1 *[(1$-$0.9999709337) + {0*$-$0.4212} + {0.9999709337-0}*{0.6236482 +

{0.079584733 * $\frac{0.000123-0}{0-0.9999709337}$}]

= 0.6236493596
