# Supplementary material for: Direct and indirect mapping of the 12-item Short Form Survey version 2 (SF-12v2) onto the EQ-5D-5L utility scores in general Thai population
Source: PLoS One. 2026 Jun 22;21(6):e0351064. doi: 10.1371/journal.pone.0351064 (PMC13286156; doi:10.1371/journal.pone.0351064)
Supplement: S2 Text — (DOCX) [file pone.0351064.s006.docx]

**S2 Text.** **Instructions for predicting utility scores using the indirect mapping algorithm**

**Example**: A 74-year-old respondent provided the following responses to the SF-12v2 items included as predictors in the direct mapping algorithm

| **Item** | **GH1** | **PF1** | **PF2** | **RP1** | **RP2** | **RE1** | **RE2** | **BP1** |
| --- | --- | --- | --- | --- | --- | --- | --- | --- |
| Original response | 4 | 1 | 1 | 3 | 3 | 2 | 2 | 3 |
| **Recode** | **GH1_r** |  |  |  |  |  |  | **BP1_r** |
| Final response | 2 | 1 | 1 | 3 | 3 | 2 | 2 | 3 |

**Step 1** Calculate subscale scores for PF, RP, BP, GH, and RE from the responses to the SF-12v2 items

PF = 0 (calculated from PF1 and PF2 where they yield 0 point)

RP = 50 (calculated from RP1 and RP2 where they yield 50 points per item)

BP = 50 (calculated from BP1 where it yields 50 points)

GH = 25 (calculated from GH1 where it yields 25 points)

RE = 25 (calculated from RE1 and RE2 where they yield 25 points per item)

**Step 2** For each dimension, calculate the estimated probabilities (*z_i_*) for problem level (*i*), where *i* = 2, 3, 4, and

5, with reference to Level 1 (the reference level) from the subscale score derived from Step 1. The coefficients can be obtained from the respective table and column for a given dimension and problem level *i*.

Let’s begin with the Mobility Dimension, the estimated probabilities are calculated as follows:

*z_2_* = -2.273 + age(0.061) + PF(-0.024) + RP(0.000) + BP(-0.004) + GH(-0.021) + RE(0.003)

= -2.273 + 74(0.061) + 0(-0.024) + 50(0.046) + 25(-0.021) + 25(0.003)

= 1.634

*z_3_* = -2.574 + age(0.082) +PF(-0.022) + RP(-0.026) + BP(-0.025) + GH(-0.027) + RE(0.013)

= -2.574 + 74(0.082) + 0(-0.022) + 50(-0.026) + 50(-0.025) + 25(-0.027) + 25(0.013)

= 0.662

*z_4_* = -15.706 + age(0.274) + PF(0.014) +RP(0.023) + BP(-0.078) + GH(-0.166) + RE(-0.026)

= -15.706 + 74(0.274) + 0(0.014) + 50(0.023) + 50(-0.078) + 25(-0.166) + 25(-0.026)

= -2.694

*z_5_* = 65.207 + age(0.205) + PF(-0.559) + RP(-2.539) + BP(-1.259) + GH(-0.098) + RE(0.024)

= 65.207 + 74(0.205) + 0(-0.559) + 50(-2.539) + BP(-1.259) + 25(-0.098) + 25(0.024)

= -111.123

**Step 3** Calculate the probability for each problem level (*i*), where *i* = 2, 3, 4, and 5 to determine the most-likely problem level for each given EQ-5D dimension. With the exception of problem level 1 (the reference level), the probability for reporting problem level *i* for each dimension can be calculated using this following formula

Pr(EQ-5D dimension for problem level (*i*)) = exp (*z_i_*)/[1 + (exp (*z_2_*) + exp (*z_3_*) + exp (*z_4_*) + exp (*z_5_*)]

For Mobility (MO) dimension, the probability of reporting each problem level (*i*) is Pr (MO = *i*):

Pr (MO = 2) = exp (1.634) / [1 + (exp (1.634) + exp (0.662) + exp (-2.694) + exp (-111.123)]

= 0.63015

Pr (MO = 3) = exp (0.662) / [1 + (exp (1.634) + exp (0.662) + exp (2.694) + exp (-111.123)]

= 0.23853

Pr (MO = 4) = exp (2.694)/ [1 + (exp (1.634) + exp (0.662) + exp (2.694) + exp (-111.123)]

= 0.00832

Pr (MO = 5) = exp (-111.123)/ [1 + (exp (1.634) + exp (0.662) + exp (2.694) + exp (111.123)]

= 0.00000

Pr (MO = 1) = 1 - 0.63015-0.23853-0.00832-0.0000

= 0.12301

Therefore, Level 2 is the most-likely problem level for the Mobility dimension because it has the highest probability of (0.63015) among all other levels (probability ranging from 0.63015 – 0.00000).

**Step 4** Repeat Step 2 and Step 3 for the rest of EQ-5D dimensions to determine the most probable problem for each EQ-5D dimension

Based on the respondent’s SF-12v2 responses, the most likely problem levels for each EQ-5D dimension are presented in the following table:

| **EQ-5D-5L dimension** | **Most-likely problem level** | **Probability** |
| --- | --- | --- |
| Mobility | 2 | 0.63015 |
| Self-care | 2 | 0.4769 |
| Usual activities | 2 | 0.6779 |
| Pain/discomfort | 2 | 0.4480 |
| Anxiety/depression | 2 | 0.4881 |

**Step 4** Convert the 5-digit health profile to the utility score using the Thai value set

The respondent’s EQ-5D-5L health profile is “22222”, which gives a utility score of 0.7030 based on the Thai value set.
