# Supplementary material for: Direct and indirect mapping of the 12-item Short Form Survey version 2 (SF-12v2) onto the EQ-5D-5L utility scores in general Thai population
Source: PLoS One. 2026 Jun 22;21(6):e0351064. doi: 10.1371/journal.pone.0351064 (PMC13286156; doi:10.1371/journal.pone.0351064)
Supplement: S1 Fig — (DOCX) [file pone.0351064.s007.docx]

**S1 Fig. Comparison of the distributions of observed and predicted utility scores across all regression models for direct and indirect mapping**

*
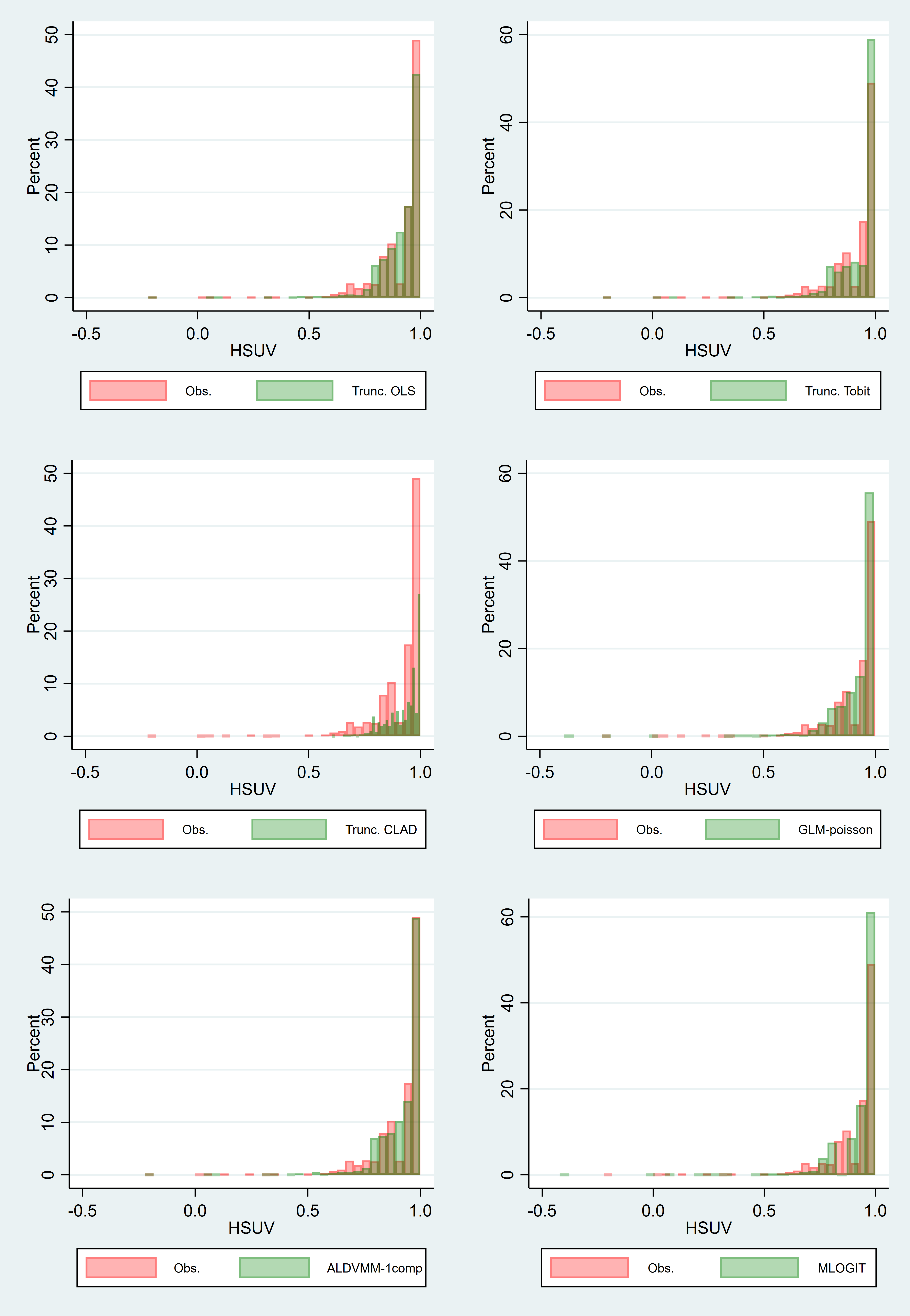
*

*ALDVMM-1 component* adjusted limited dependent variable mixture model with 1 component *CLAD* censored least absolute deviation *GLM* generalized linear model *MLOGIT* multinomial logistic regression *OLS* ordinary least squares *Trunc* truncated predicted utility values for the OLS, Tobit, and CLAD. Their plots were generated with predicted values truncated at theoretical boundaries of the Thai EQ-5D-5L value set: an upper bound of 1.0 for values exceeding 1 and a lower bound of –0.4212 for values less than –0.4212. Truncation did not affect the comparative assessment of model performance. Non-truncated predictions are shown for GLM-poisson, ALDVMM-1 component, and MLOGIT, which generate predicted utility values within the Thai-specific value set by model structures.
